# Precancerous lesion determinants in women attending cervical cancer screening at public health facilities in North Shoa Zone, Amhara, Ethiopia: an unmatched case-control study

**DOI:** 10.1186/s12905-024-03113-z

**Published:** 2024-05-03

**Authors:** Dereje Abebe Teklehaimanot, Abinet Dagnaw Mekuria, Abel Fekadu Dadi, Behailu Tariku Derseh

**Affiliations:** 1 Debre Berhan Comprehensive Specialized Hospital, Debre Berhan, Ethiopia; 2 Department of Public Health, Asrat Woldeyes Health Sciences Campus, Debre Berhan University, Debre Berhan, Ethiopia; 3 Addis Continental Institute of Public Health, Addis Ababa, Ethiopia; 4 Menzies School of Health Research, Charles Darwin University, Darwin, NT Australia

**Keywords:** Pre-cancerous lesions, Sexually transmitted diseases, Oral contraceptives

## Abstract

**Background:**

Precancerous cervical lesions develop in the transformation zone of the cervix and progress through stages known as cervical intraepithelial neoplasia (CIN) 1, 2, and 3. If untreated, CIN2 or CIN3 can lead to cervical cancer. The determinants of cervical precancerous lesions are not well documented in Ethiopia. Therefore, this study aims to find the determinants of cervical precancerous lesions among women screened for cervical cancer at public health facilities.

**Methods:**

A study conducted from January to April 2020 involved 216 women, consisting of 54 cases (positive for VIA during cervical cancer screening) and 162 controls (negative for VIA). It focused on women aged 30 to 49 undergoing cervical cancer screening. Multivariable logistic regression analysis assessed the link between precancerous lesions and different risk factors, considering a significance level of *p* < 0.05.

**Results:**

Women who used oral contraceptives for a duration exceeding five years showed a nearly fivefold increase in the likelihood of developing precancerous lesions (Adjusted Odds Ratio (AOR) = 4.75; 95% CI: 1.48, 15.30). Additionally, early age at first sexual intercourse (below 15 years) elevated the odds of developing precancerous lesions fourfold (AOR = 3.77; 95% CI: 1.46, 9.69). Furthermore, women with HIV seropositive results and a prior history of sexually transmitted infections (STIs) had 3.4 times (AOR = 3.45; 95% CI: 1.29, 9.25) and 2.5 times (AOR = 2.58; 95% CI: 1.10, 6.09) higher odds of developing cervical precancerous lesions compared to their counterparts.

**Conclusion:**

In conclusion, women who have used oral contraceptives for over five years, started sexual activity before the age of 15 and have a history of sexually transmitted infections, including HIV, are at higher risk of developing precancerous cervical lesions. Targeted intervention strategies aimed at promoting behavioural change to prevent early sexual activity and STIs are crucial for avoiding cervical precancerous lesions. It is crucial to introduce life-course principles for female adolescents early on, acknowledging the potential to prevent and control precancerous lesions at critical stages in life, from early adolescence to adulthood, encompassing all developmental phases.

**Supplementary Information:**

The online version contains supplementary material available at 10.1186/s12905-024-03113-z.

## Background

Precancerous cervical lesions develop in the transformation zone of the cervix and progress through stages known as cervical intraepithelial neoplasia (CIN) 1, 2, and 3. If untreated, CIN2 or CIN3 can lead to cervical cancer [1–4]. Early detection and treatment of precancerous lesions through methods like cryotherapy, loop electrosurgical excision procedures, and laser treatment can prevent cervical cancer [3–7]. Additionally, WHO-approved HPV vaccines, administered widely in wealthier countries, play a crucial role in prevention, although access remains limited in Africa, where the cervical cancer burden is high [2, 3, 8].

Cervical cancer arises in the cervical region of females, typically progressing from an extended period of pre-invasive cervix lesions [9]. Usually, Human Papilloma Virus (HPV) is associated with temporary infections or mild, self-limiting lesions. However, persistent HPV infection can progress to precancerous and cancerous lesions in different areas of the body [10–14]. Globally, cervical cancer is responsible for an estimated 528,000 new cancer cases and 266,000 deaths [4]. Factors contributing to its occurrence include high-risk sexual behaviour, smoking, excessive alcohol use, family history, and individuals with compromised immune systems. However, numerous studies emphasise that the most common cause of cervical cancer is viral origin, particularly HPV [15]. Notably, cervical cancer is highly preventable and manageable through early screening during the pre-invasive stage, mass immunization against HPV before the initiation of sexual activity, and the introduction of effective treatments. These measures can significantly benefit individuals and reduce the burden of morbidity and mortality associated with cervical cancer [16–18].

In sub-Saharan Africa, the prevalence of cervical cancer ranges from 7.5 to 10% [8]. In Ethiopia, the risk of cervical cancer was significant in 2019, with an estimated incidence of 6570 [95% UI 4470–106,400] and 3870 [95% UI 2680–6290] deaths [19]. Cervical cancer is entirely preventable and treatable through measures such as mass vaccination against HPV during childhood, regular screening for sexually active women, and timely diagnosis and treatment of pre-symptomatic cases. However, at an advanced stage, the disease becomes challenging to cure, incurring high costs and often resulting in fatal outcomes [20]. Despite its substantial impact, there is a lack of information, limited screening facilities, insufficiently trained health workers, and insufficient attention from policymakers, program managers, health facilities, stakeholders, communities, and families [21, 22].

Visual Inspection with Acetic Acid (VIA) is a screening method for cervical precancerous lesions. A sound understanding of women’s pelvic anatomy and the natural progression of cervical cancer equips healthcare providers with the knowledge to effectively communicate and enhance awareness of cervical cancer prevention among women, families, and communities [23]. Ethiopia’s national guidelines for cervical cancer prevention, initially issued in 2015 and revised in 2021, provide screening and treatment procedures directives. Visual Inspection with Acetic Acid (VIA) is favoured as the screening method in settings with limited resources due to its cost-effectiveness compared to HPV testing or Pap smears [20, 24, 25]. VIA is utilized for detecting cervical precancerous lesions. Healthcare providers equipped with comprehensive knowledge of women’s pelvic anatomy and the natural progression of cervical cancer can effectively communicate and raise awareness about cervical cancer prevention among women, families, and communities [23]. The National Cancer Control Plan emphasizes cancer prevention, early detection, and treatment, including initiatives such as HPV vaccination for girls before sexual activity and the expansion of cancer treatment services. Ethiopia is dedicated to advancing cancer prevention, early detection, and improving diagnosis and treatment. The strategy also emphasizes the importance of cancer research in achieving these objectives [20].

While we know precancerous cervical lesions’ prevalence and associated risk factors, more evidence should be needed to address area-specific determinant factors. National strategies acknowledge the need for research to bridge this gap, emphasizing more localized evidence to inform effective interventions and policies. Thus, urgent research initiatives are needed to fill this gap and provide comprehensive insights into precancerous cervical lesions’ determinant factors at the local level, enabling tailored strategies for prevention and management. Hence, this study aimed to pinpoint predictors of precancerous cervical lesions supporting the national plan.

## Methods

### Study design and setting

We undertook a multi-center, unmatched case-control study to investigate the factors influencing pre-cancerous lesions in women undergoing cervical cancer screening services from January to April 2020. In this study, eight health facilities, namely Debre Berhan Comprehensive Specialized Hospital, Enat Hospital, Mehal-Meda Hospital, Debre Berhan 04 Health Centre (HC), Shewarobit HC, Debre-Sina HC, Gorebela HC, and Keyit HC were included in the analysis. A multi-disciplinary health team provides medical services, including Nurses, midwives, general practitioners, gynecologists, pediatricians, emergency surgeons, and other paramedics. These health facilities are meant to provide community preventive, curative and rehabilitative services. These health facilities provide medical services for over two million people in the catchment area. Health facilities offer outpatient, in-patient, and rehabilitation services 24 h a day, seven days a week. The sample required for this study was randomly selected from these health facilities. Three hospitals and five health centres were chosen as the primary facilities providing pre-cervical cancer screenings. Cases and controls were identified during the screening process.

### Outcome variable and selection of study participants

The study’s outcome was pre-cancerous cervical lesions, characterized by abnormal cell growth on the cervix’s surface, which can occur at any of the three stages [24]. All women aged 30–49 who underwent screening for cervical precancerous lesions were included in the study. However, women meeting any of the following criteria were excluded: those with suspicious lesions detected via visual inspection with acetic acid (VIA), individuals experiencing menstrual periods at the time of the study, and pregnant women. Cases included women aged 30–49 who participated in pre-cervical lesion screening, tested positive for Visual Inspection with Acetic Acid (VIA) during the study period and were free of suspicious lesions with VIA. Additionally, cases were not menstruating and were not pregnant at the time of the study. Controls comprised women aged 30–49 years who underwent pre-cervical lesion screening and tested negative for VIA during the study period [24].

The screening was performed by trained healthcare professionals, including nurses, midwives, physicians, health officers, gynecologists, obstetricians, and emergency surgeons. These providers applied diluted acetic acid (3 to 5%) to the cervix and waited one minute to determine the result. If the acetic acid caused a temporary white appearance (aceto-white) on the cervix, it was immediately assessed as VIA positive. Conversely, smooth and uniformly pink features on the cervix indicated a negative result. Sociodemographic, reproductive, maternal lifestyle and behavioural factors were collected and examined as potential determinants of positive pre-cancerous cervical lesions.

### Sample size determination

We calculated the sample size using Epi Info™ Team (Epi Info 7.2.0.1, 2016) with the following assumptions: a two-sided confidence level of 95%, a power of 80%, a case-to-control ratio of 1:3, a percentage of controls exposed at 36.68%, an odds ratio of 2.55, and a percentage of cases with exposure at 61.4% [26]. Based on these parameters, 49 cases and 147 controls were taken to make the total sample size of 196. After accounting for non-response, the study involved 216 participants, with a non-response rate of 10%.

### Data collection tool and procedure

The data was collected using a structured interviewer-administered questionnaire designed for this study (Additional file 1). The tool has socio-demographic and economic characteristics, reproductive health-related information, and information on lifestyle and sexual behaviour. The questionnaire was prepared in English and translated into the local language (Amharic version). The questionnaire was back-translated to English to check for consistency with the English version. Eight trained Midwifery nurses collected the data in collaboration with eight senior and trained professionals. The principal investigator gave one day of training to both data collectors and supervisors. A senior BSc midwifery professional supervised and monitored the data collection processes.

### Data collection and analysis

Before the data collection, a pretest was conducted on 5% of the sample. The collected data were thoroughly checked for completeness, entered Epi-Data version 3.1, and subsequently exported to SPSS version 20 for analysis. Data management was performed to facilitate the data preparation for analysis, involving frequency distribution tables, graphic representations, and histograms. Descriptive statistics, including percentages, means, and standard deviations, were applied to most variables in the study, encompassing socio-demographic and behavioural factors.

We divide the wealth index data into quartiles: Q1 (25th percentile), Q2 (50th percentile or median), and Q3 (75th percentile). Then, we establish thresholds for “poor” and “rich” wealth index categories. In this study, individuals with incomes below the median are considered “poor,” while those above the median are considered “rich.”

We took all variables with p-value < 0.2 into the multivariable model to minimize the effects of possible confounders. We checked for multicollinearity of independent factors using variance inflation factor (VIF) > 10. We found the model discrimination capacity to be good with the Hosmer-Lemeshow goodness p-value of 0.933. Frequency, percentage, texts, and table presented the descriptive findings. The odds ratio and a 95% Confidence Interval (CI) were estimated to measure the determinants of pre-cancerous cervical lesions using multivariable logistic regression. Variables significant at a p-value less than 0.05 were considered an essential determinant of the pre-cancerous cervical lesion.

## Results

### Socio-demographic characteristics

The average age of respondents was 38.28 years, with a standard deviation of ± 5.85. Most controls (61.1%) and cases (63.0%) fell within the age range of 30–39 years. A significant proportion of controls (27.8%) and cases (31.5%) could not read and write. Most controls (69.1%) and cases (77.8%) resided in urban areas (Table 1).

**Table 1:** Socio-demographic characteristics of study participants in North Shoa, Ethiopia, 2020

Variables	Cases, *N* (%)	Controls, *N* (%)
Age		
30–39 years 40–49 years	34(63.0)	99(61.1)
	20(37.0)	63(38.9)
Level of education		
I can’t read and write. Primarily (1–8) Secondary (9–12) Diploma and Above	17(31.5)	45(27.8)
	16(29.6)	42(25.9)
	11(20.4)	32(19.8)
	10(18.5)	43(26.5)
Body mass index		
< 18.5 18.5–24.9 25–30	4(7.4)	14(8.6)
	29(53.7)	103(63.6)
	21(38.9)	45(27.8)
Wealth index		
Poor Rich	28(51.9)	105(64.8)
	26(48.1)	57(35.2)
Marital status		
Single Married Widowed Divorced	4(7.4)	14(8.6)
	40(74.1)	125(77.2)
	4(7.4)	11(6.8)
	6(11.1)	12(7.4)
Occupation		
Housewife Worker	31(57.4)	71(43.8)
	23(42.6)	91(56.2)
Place of residence		
Urban Rural	42(77.8)	112(69,1)
	12(22.2)	50(30.9)

### Reproductive health history

The prevalence of oral contraceptive use was 112 (69.1%) among controls and 41 (75.9%) among cases. Seventy-seven (47.5%) controls and 32 (59.3%) cases experienced their first menarche before age 14. A significant majority, 144 (88.9%) of controls and 47 (87%) of cases had a history of childbirth, with 144 (88.9%) of controls and 47 (87%) of cases having less than five children. Most controls (84.6%) and cases (96.3%) had their first childbirth between the ages of 15 and 24 years (Table 2).

**Table 2:** Reproductive history among study participants in health facilities, Ethiopia, 2020

Variables	Cases, *N* (%)	Controls, *N* (%)
Ever used contraceptives in any method.		
No Yes	7(13)	22(13.6)
	47(87)	140(86.4)
How long have you used oral contraceptives?		
No < 5yrs *>*5yrs	13(24.1)	50(30.9)
	27(50)	102(63.0)
	14(25.9)	10(6.2)
Ever used IUCD		
No Yes	14(25.)	73(45.1)
	40(74.1)	89(54.9)
How long used IUCD		
No < 5yrs ≥ 5yrs	14(25.9)	73((45.1)
	20(37)	37(22.8)
	20(37)	52(32.1)
Pattern of menstrual history		
Regular Irregular	32(59.3)	111(68.5)
	22(40.7)	51(31.5)
Post-coital bleeding		
No Yes	35(64.8)	123(75.9)
	19(35.2)	39(24.1)
Ever give birth		
No Yes	1(1.9)	6(3.7)
	53(98.1)	156(96.3)
Age at first birth		
15-24yrs 25-34yrs	35(29.7)	82(87.3)
	18(23.3)	74(68.7)
Genital trauma at the time of delivery		
No Yes	35(64.8)	122(75.3)
	19(35.2)	40(24.7)
Type of trauma		
No Trauma Cervix Vagina Both	35(64.8)	122(75.3)
	4(7.4)	11(6.8)
	9(16.7)	20(12.3)
	6(11.1)	9(5.6)
Average birth interval		
No 1-2yrs ≥2yrs	4(7.4)	37(37)
	29(53.7)	67(41.4)
	21(38.9)	58(35.8)
History of Abortion		
No Yes	18(33.3)	82(50.6)
	36(66.7)	80(49.4)
Family history of cervical cancer		
No Yes	49(90.7)	153(94.4)
	5(9.3)	9(5.6)

### Lifestyle and sexual behavior

Half, 84 (51.9%) of controls and 40 (74.1%) of cases had a history of sexually transmitted infections. In many cases, 45 (83.3%) and 114 (70.4%) controls had developed pelvic infection. Twenty-six (16%) of controls and 19 (35.2%) of cases had started first sexual intercourse at the age of less than 15 years. More than two-thirds, 117 (72.2%) of the controls and 42 (77.8%) of the cases, had never used a condom during sexual intercourse. About 12(7.4) of the controls and 12(22.25) of the cases are HIV positive (Table 3).

**Table 3:** Lifestyle and sexual behaviour of study participants in North Shoa, Ethiopia, 2020

Variables	Cases, *N* (%)	Controls, *N* (%)
Ever screened for cervical cancer.		
No Yes	51(94.4)	150(92.6)
	3(5.6)	12(7.4)
Ever smoked		
No Yes	53(98.1)	159(98.1)
	1(1.9)	3(1.9)
Age at first sexual intercourse		
<15yrs 15-17yrs >18yrs	19(35.2)	26(16)
	20(37)	46(28.4)
	15(27.8)	90(55.6)
Condom used during sexual intercourse		
No Yes	42(77.8)	117(72.2)
	12(22.2)	45(27.8)
Pelvic infection		
No Yes	9(16.7)	48(29.6)
	45(83.3)	114(70.4)
History of STI		
No Yes	14(25.9)	78(48.1)
	40(74.1)	84(51.9)
History of genital ulcer or swelling		
No Yes	43(79.6)	122(75.3)
	11(20.4)	40(24.7)
Partner’s history of genital ulcer		
No Yes	37(68.5)	127(78.4)
	17(31.5)	35(21.6)
HIV status		
Reactive Non-reactive Undetermined	12(22.2)	12(7.4)
	41(75.9)	134(82.7)
	1(1.9)	16(9.9)
Partners have other partners.		
No Yes	16(19.6)	53(32.7)
	38(70.4%)	109(67.3)

### Determinants of pre-cancerous cervical lesions among study participants

Multivariate analysis found the use of oral contraceptives for more than five years (Adjusted Odds Ratio (aOR) = 4.75; 95% CI: 1.48, 15.30), age of first sexual intercourse at less than 15 years (aOR = 3.77; 95% CI: 1.46, 9.69), history of sexually transmitted infections (aOR = 2.63; 95%CI: 1.15, 6.00), and HIV positive (aOR = 3.45; 95% CI: 1.23, 9.25) were the determinants of pre-cancerous cervical lesions at p-value < 0.05 (Table 4).

**Table 4:** Bivariable and Multivariable analysis of the determinants of pre-cancerous lesions among study participants in North Shoa health facilities, Ethiopia, 2020

Variables	Controls, *N* (%)	Cases, *N* (%)	cOR (95%CI)	aOR (95% CI)
Age of first marriage				
<18yrs ≥18yrs	86(53.1)	35(64.8)	1.63(0.86, 3.08)	1.87 (0.88, 3.95)
	76(46.9)	19(35.2)	1	1
Use of oral contraceptive				
No < 5yrs >5yrs >15yrs	50(30.9)	13(24.1)	1	1
	102(63.0)	27(50)	1.02 (0.48, 2.14)	1.01 (0.42, 2.42)
	10(6.2)	14(25.9)	5.38 (1.95, 14.86)	4.75 (1.47,15.30)*
	85(52.5)	22(40.7)	1	1
Age of first birth				
15-24yrs 25-34yrs	82 (87.3)	35 (29.7)	0.57(0.29, 1.09)	0.78(0.67, 1.68)
	74 (68.7)	18 (23.3)	1	1
Average birth interval				
No 1-2yrs ≥2yrs	37(37)	4(7.4)	1	1
	67(41.4)	29(53.7)	4.01(1.31, 12.27)	1.75 (0.42, 7.27)
	58(35.8)	21(38.9)	3.349(1.06, 10.54)	1.61(0.36,7.27)
History of abortion				
No Yes	82(50.6)	18(33.3)	1	1
	80(49.4)	36(66.7)	2.05(1.07,3.91)	1.57(0.67, 3.67)
Age of first sexual intercourse				
<15yrs 15-17yrs >18yrs	26(16)	19(35.2)	4.38(1.96, 9.81)	3.77(1.46, 9.69)*
	46(28.4)	20(37)	2.61(1.22, 5.57)	2.58(1.09, 6.08)*
	90(55.6)	15(27.8)	1	1
Pelvic Infection				
No Yes	48(29.6)	9(16.7)	1	1
	114(70.4)	45(83.3)	2.11(0.95, 4.64)	0.76(0.22, 2.73)
Sexually Transmitted Infection				
No Yes	78(48.1)	14(25.9)	1	1
	84(51.9)	40(74.1)	2.65(1.34, 5.25)	2.63(1.15, 6.01)*
The result HIV				
Positive Unknown Negative	12(7.4)	12(22.2)	3.27(1.36, 7.83)	3.45 (1.29, 9.25)*
	16(9.9)	1(1.9)	0.20 (0.03, 1.58)	0.43(0.05, 3.75)
	134(82.7)	41(75.9)	1	1

Note: *indicates significant variables at p-value < 0.05; cOR means Crude Odds Ratio; aOR means Adjusted Odds Ratio

## Discussion

Early detection and treatment of pre-cancerous cervical lesions is critical to prevent the progression of cervical cancer. We developed a model that would help clinicians prioritize women at higher risk of developing pre-cancerous cervical lesions for screening. These women are those who have used oral contraceptives for more than five years, had a history of early (before age 15 years) initiation of sexual intercourse, and had sexually transmitted infections, including HIV/AIDS.

This study shows that women who have used oral contraceptives for an extended period, specifically more than five years, have significantly higher odds of developing precancerous cervical lesions compared to those who have not used oral contraceptives. Similar results from studies from Ethiopia [27], Indonesia [28], and Thailand [29] support this finding. One potential explanation for this association is that prolonged use of oral contraceptive hormones may lead to changes in the cervical tissue. Specifically, it may cause the columnar epithelium found within the cervical canal to protrude or “evert” onto the surface of the cervix (ectocervix) [30]. This exposure of the columnar epithelium to the surface of the cervix could potentially increase the susceptibility to Human Papillomavirus (HPV) infection. Human papillomavirus (HPV) infection is a well-established risk factor for the development of cervical lesions and cervical cancer. Therefore, the increased exposure of the columnar epithelium to HPV due to eversion may contribute to the higher risk of precancerous lesions among women who have used oral contraceptives for an extended period.

This study also indicates that early initiation of sexual intercourse is associated with an increased risk of developing precancerous cervical lesions. Our analysis revealed that women who initiated sexual intercourse before the age of 15 were three times more likely to develop precancerous cervical lesions compared to those who began sexual activity after the age of 18. This finding is supported by research conducted in Ethiopia [27, 31] and Rwanda [32], further validating the relationship between early sexual initiation and cervical health outcomes. Furthermore, our study found that women who initiated sexual intercourse between the ages of 15 and 17 had twice the odds of developing precancerous cervical lesions compared to those who started after the age of 18. This evidence aligns with previous research conducted in Ethiopia, highlighting the consistent association between early sexual debut and cervical health risks [33].

The increased risk associated with early sexual initiation can be due to various factors. Firstly, engaging in sexual activity at a young age often involves partners who may have had multiple sexual encounters, thereby increasing the likelihood of exposure to sexually transmitted infections (STIs), including human papillomavirus (HPV). Additionally, the cervix of adolescents is biologically immature and more susceptible to HPV infection, potentially contributing to the development of precancerous lesions.

Sexually Transmitted Infections (STIs) represent another significant determinant factor for the development of precancerous cervical lesions among women, as evidenced by our study. Women with a history of STIs were found to have 2.6 times higher odds of developing precancerous cervical lesions compared to those without such a history. This finding aligns with previous research in Ethiopia [22, 34], Egypt [35], and Morocco [36], indicating a consistent association between STIs and precancerous lesions. One potential explanation for this association is the co-infection of HPV with other STIs. The presence of additional STIs may exacerbate the effects of HPV infection, leading to an increased risk of precancerous cervical lesions. Furthermore, our study observed a heightened risk of precancerous lesions among HIV-positive women compared to HIV-negative individuals. This co-infection underscores the importance of regular cervical cytological examination for immunocompromised women, as they may be more susceptible to HPV infection and subsequent development of precancerous lesions.

Moreover, our findings highlight the need to address low intention and uptake of cervical cancer screening among women. Previous studies have indicated a need for more awareness or motivation regarding screening, posing challenges to early detection and prevention efforts [37–39]. This low intention underscores the importance of targeted interventions to enhance screening intentions and uptake among women, particularly in resource-limited settings. The observed association between immune suppression, HPV infection, and the development of precancerous cervical lesions suggests the need for comprehensive approaches to cervical cancer prevention. Efforts to improve immune function and reduce susceptibility to HPV infection through vaccination and other preventive measures are crucial in mitigating the risk of precancerous lesions and cervical cancer development.

The implications of this research underscore the imperative of comprehensive strategies focusing on screening, early detection, and treatment of precancerous cervical lesions, alongside addressing sexually transmitted infections (STIs), including HIV, and prioritizing HPV vaccination. According to national and global strategies for the control and prevention of cervical cancer, prioritizing regular screening programs for precancerous cervical lesions is crucial in identifying and treating abnormalities before they progress to cancer. Early detection not only improves treatment outcomes but also reduces the burden of advanced cervical cancer cases.

Addressing STIs, particularly Human Papillomavirus (HPV) infection, is paramount in preventing the development of precancerous lesions [33]. Given the strong association between persistent HPV infection and cervical cancer, stakeholders should strive towards promoting safe sexual practices, increasing awareness about HPV, and ensuring access to HPV vaccination [40], especially among young individuals. HPV vaccination plays a pivotal role in preventing HPV infection and subsequently reducing the risk of precancerous lesions and cervical cancer. Efforts should focus on enhancing vaccine coverage, especially in high-risk populations and resource-limited settings.

Moreover, recognizing the heightened risk of cervical cancer among individuals with persistent HPV infection and immunocompromised individuals, such as those living with HIV, underscores the importance of tailored screening and treatment interventions. Clinicians and health facility leaders should prioritize regular cervical cancer screening and receive appropriate medical care to mitigate the risk of progression to cervical cancer.

The study’s robustness lies in its rigorous quality assurance measures, meticulous data collection procedures, and inclusion of all health facilities offering screening services in the catchment area. Consequently, these factors enhance the potential generalizability of the findings to a broader population. However, readers should interpret the results of this study by considering the following limitations. Some participants may keep their problem private during history taking and data collection from cultural and reduced literacy. However, to minimize underestimation of the findings, data collectors were previously trained intensively for ten days to gather relevant information by explaining the purpose of the study and providing genuine answers. Consequently, data collectors use the standard technique to reduce the recall and social desirability bias that could arise during data collection.

## Conclusions

In conclusion, women who have used oral contraceptives for over five years initiated sexual activity before the age of 15, and have a history of sexually transmitted infections, including HIV, are at higher risk of developing precancerous cervical lesions. Targeted intervention strategies aimed at promoting behavioural change to prevent early sexual activity and STIs are crucial for avoiding cervical precancerous lesions. Introducing life-course principles to female adolescents early on can help prevent and control diseases at critical developmental stages. It is essential to prioritize comprehensive sexual education and access to reproductive health services, particularly for adolescents, by educating them about the risks of early sexual debut and promoting preventive measures like HPV vaccination and regular cervical cancer screening. Efforts to delay sexual initiation and encourage healthy sexual behaviours can significantly reduce the incidence of cervical cancer in the long term.

### Electronic supplementary material

Below is the link to the electronic supplementary material.

Additional file 1: English version of the questionnaire

## Data Availability

All data generated or analyzed during this study is available in the article.

## Abbreviations

AOR: Adjusted Odds Ratio
ART: Anti-Retroviral Therapy
CDC: Centre for Disease Control
CI: Confidence Interval
FMOH: Federal Ministry of Health
HIV: Human Immunodeficiency Virus
HPV: Human Papilloma Virus
ICC: Invasive Cervical Cancer
NCCP: National Cancer Control Plane
OR: Odds Ratio
STI: Sexual Transmitted Infection
SPSS: Statistical Package of Social Science
VIA: Visual Inspection with Acetic Acid
WHO: World Health Organization

## References

[1] Canadian Cancer Society. Precancerous conditions of the cervix. 2019.

[2] World Health Organization. Guidelines for screening and treatment of precancerous lesions for cervical cancer prevention. WHO Guidel. 2013;:60. 24716265

[3] WHO. Cervical cancer: Fact sheets. 2022.

[4] Asseffa NA. Cervical Cancer: Ethiopia´s Outlook. J Gynecol Women’s Heal. 2017;5.

[5] Pathfinder international. Single-Visit Approach to Cervical Cancer Prevention. Clinical Standards of Practice and Counseling Guidelines. 2012.

[6] Mishra GA, Pimple SA, Shastri SS (2011). An overview of prevention and early detection of cervical cancers. Indian J Med Paediatr Oncol.

[7] Wright TC, Massad LS, Dunton CJ, Spitzer M, Wilkinson EJ, Solomon D (2007). 2006 consensus guidelines for the management of women with abnormal cervical cancer screening tests. Am J Obstet Gynecol.

[8] Bruni L, Barrionuevo-Rosas L, Albero G, Serrano B, Mena M, Gómez D et al. Human Papillomavirus and Related Diseases Report in the World: Summary Report. 2017.

[9] Reddy KS (2016). Global burden of Disease Study 2015 provides GPS for global health 2030. Lancet.

[10] Naucler P, Ryd W, Törnberg S, Strand A, Wadell G, Elfgren K (2007). Human papillomavirus and papanicolaou tests to screen for cervical Cancer. N Engl J Med.

[11] Lei J, Ploner A, Elfström KM, Wang J, Roth A, Fang F (2020). HPV Vaccination and the risk of Invasive Cervical Cancer. N Engl J Med.

[12] Giannini A, Di Donato V, Sopracordevole F, Ciavattini A, Ghelardi A, Vizza E (2023). Outcomes of high-Grade cervical dysplasia with positive margins and HPV persistence after cervical conization. Vaccines.

[13] Bogani G, Sopracordevole F, Ciavattini A, Ghelardi A, Vizza E, Vercellini P, et al. HPV-related lesions after hysterectomy for high-grade cervical intraepithelial neoplasia and early-stage cervical cancer: a focus on the potential role of vaccination. Tumori. 2023. 3008916231208344. 10.1177/03008916231208344 37978580

[14] Bogani G, Sopracordevole F, Ciavattini A, Vizza E, Vercellini P, Giannini A (2023). Duration of human papillomavirus persistence and its relationship with recurrent cervical dysplasia. Eur J Cancer Prev.

[15] Momenimovahed Z, Salehiniya H (2017). Incidence, mortality and risk factors of cervical cancer in the world. Biomed Res Ther.

[16] Botelho MC, Alves H, Richter J, Diseases C, Herculano RA, Health I. HHS Public Access. 2017;14 Estrogen catechols detection as biomarkers in schistosomiasis induced cancer and infertility:135–8. 10.2174/1570180813666160720165057 PMC5179139 28018158

[17] Converse PJ, Mariam DH, Mulatu M, Mekonnen W, Kloos H. Bibliography on HIV / AIDS in Ethiopia and Ethiopians in the Diaspora: The 2012 Update. 2012.

[18] Bogani G, Raspagliesi F, Sopracordevole F, Ciavattini A, Ghelardi A, Simoncini T et al. Assessing the long-term role of vaccination against HPV after Loop Electrosurgical Excision Procedure (LEEP): a propensity-score matched comparison. Vaccines. 2020;8. 10.3390/vaccines8040717 PMC7711506 33271963

[19] Awedew AF, Asefa Z, Belay WB (2022). National Burden and Trend of Cancer in Ethiopia, 2010–2019: a systemic analysis for global burden of disease study. Sci Rep.

[20] Federal Ministry of Health, Ethiopia. National Cancer Control Plan 2016–2020. Addis Ababa; 2015.

[21] Ntekim A. Cervical Cancer in Sub Sahara Africa. Topics on Cervical Cancer with an advocacy for Prevention. InTech; 2012.

[22] Tadesse SK. Preventive mechanisms and treatment of Cervical Cancer in Ethiopia. Gynecol Obstet. 2015;5.

[23] WHO. Compr Cerv Cancer Control Geneva. 2014;:366–78.

[24] Federal Democratic Republic of Ethiopia. Guideline for Cervical Cancer Prevention and Control in Ethiopia. 2019.

[25] Federal democratic republic of Ethiopia. Ministry of Health. (2021) Guideline for Cervical Cancer Prevention and Control in Ethiopia. 2021.

[26] Teame H, Addissie A, Ayele W, Hirpa S, Gebremariam A, Gebreheat G et al. Factors associated with cervical precancerous lesions among women screened for cervical cancer in Addis Ababa, Ethiopia: a case-control study. 2018;39:1–13. 10.1371/journal.pone.0191506 PMC5774809 29352278

[27] Kassa RT (2018). Risk factors associated with precancerous cervical lesion among women screened at Marie Stops Ethiopia, Adama town, Ethiopia 2017: a case-control study. BMC Res Notes.

[28] Wahidin M, Djuwita R, Adisasmita A. Oral contraceptive and breast Cancer risks: a case-control study in six Referral hospitals in Indonesia. 2018;19:2199–203. 10.22034/APJCP.2018.19.8.2199 PMC6171374 30139225

[29] Marks MA, Gupta S, Liaw KL, Tadesse A, Kim E, Phongnarisorn C (2015). Prevalence and correlates of HPV among women attending family-planning clinics in Thailand. BMC Infect Dis.

[30] Bright PL, Norris Turner A, Morrison CS, Wong EL, Kwok C, Yacobson I (2011). Hormonal contraception and area of cervical ectopy: a longitudinal assessment. Contraception.

[31] Getinet M, Gelaw B, Sisay A, Mahmoud EA, Assefa A (2015). Prevalence and predictors of pap smear cervical epithelial cell abnormality among HIV-positive and negative women attending gynecological examination in cervical cancer screening centre at Debre Markos referral hospital, East Gojjam, Northwest Ethiopia. BMC Clin Pathol.

[32] Makuza JD, Nsanzimana S, Muhimpundu MA, Pace LE, Ntaganira J, Riedel DJ (2015). Prevalence and risk factors for cervical cancer and pre-cancerous lesions in Rwanda. Pan Afr Med J.

[33] Hailemariam T, Yohannes B, Aschenaki H, Mamaye E, Orkaido G, Seta M (2017). Prevalence of Cervical Cancer and Associated Risk factors among women attending cervical Cancer screening and Diagnosis Center at Yirgalem General Hospital, Southern Ethiopia. J Cancer Sci Ther.

[34] Geremew AB, Gelagay AA, Azale T (2018). Comprehensive knowledge on cervical cancer, attitude towards its screening and associated factors among women aged 30–49 years in Finote Selam town, northwest Ethiopia. Reprod Health.

[35] El-Moselhy EA, Altam HMB (2020). Cervical Cancer: Sociodemographic and clinical risk factors among adult Egyptian females. J Oncol Res Treat.

[36] Berraho M, Amarti-Riffi A, El-Mzibri M, Bezad R, Benjaafar N, Benideer A (2017). HPV and cofactors for invasive cervical cancer in Morocco: a multicentre case-control study. BMC Cancer.

[37] Temesgen K, Workie A, Dilnessa T, Abate M (2019). Proportions of pre-cancerous cervical lesions and its Associated factors among women clients in the Age Group of 30-49yrs in Gynecology Ward of Dessie Referral Hospital and FGAE, North-East Ethiopia, 2016. J Cancer Tumor Int.

[38] Chambuso RS, Shadrack S, Lidenge SJ, Mwakibete N, Medeiros RM (2017). Influence of HIV/AIDS on Cervical Cancer: a retrospective study from Tanzania. J Glob Oncol.

[39] Adjorlolo-Johnson G, Unger ER, Boni-Ouattara E, Touré-Coulibaly K, Maurice C, Vernon SD (2010). Assessing the relationship between HIV infection and cervical cancer in Côte d’Ivoire: a case-control study. BMC Infect Dis.

[40] Burger EA, Portnoy A, Campos NG, Sy S, Regan C, Kim JJ (2021). Choosing the optimal HPV vaccine: the health impact and economic value of the nonavalent and bivalent HPV vaccines in 48 Gavi-eligible countries. Int J Cancer.

