# Supplementary material for: Precancerous lesion determinants in women attending cervical cancer screening at public health facilities in North Shoa Zone, Amhara, Ethiopia: an unmatched case-control study
Source: BMC Womens Health. 2024 May 3;24:271. doi: 10.1186/s12905-024-03113-z (PMC11067165; doi:10.1186/s12905-024-03113-z)
Supplement: Supplementary file 1 — Additional file 1: English version of the questionnaire [file 12905_2024_3113_MOESM1_ESM.pdf]

ANNEX IV: English version questionnaire.

A questionnaire on the assessment of the determinants of precancerous lesions and associated factors among women who attend cervical cancer screenings in the Amhara region, north Shoa zone, and governmental health facility in Ethiopia.

Part 1: socio-demographic characteristics

S.N.	Question	Response	Skip
101	How old are you? complete year	________________	
102	What is your level of education	1. Don't Wright and read.<br>2. Primarily (1-8)<br>3. Secondary (9-12)<br>4. Diploma or vocational/technical<br>5. Higher/bachelor's degree and above	
103	Weight	__________kg	
104	Height	_____________cm	
105	What is your marital status?	1. Single<br>2. Married<br>3. Widowed<br>4. Divorced<br>5. Separated	
106	How old were you when you first married? (If she is married once)	__________________	
107	What is your current occupational status?	1. Housewife<br>2. Merchant<br>3. Daily labourer<br>4. Governmental employer<br>5. Private /NGO employs<br>6. Other / specify----------	
108	What is your place of residence?	1. Urban<br>2. Rural	

Household socio-economic status (Wealth Index).

No	Questions	Response	Skip
109	Do you have a television?	1. Yes 2. No	
110	Do you have a radio/tape recorder?	1. Yes 2. No	
111	Do you have a mobile telephone?	1. Yes 2. No	
112	Do you have a non-mobile/fixed telephone?	1. Yes 2. No	
113	Do you have an electric stove?	1. Yes 2. No	
114	Do you have a sofa?	1. Yes 2. No	
115	Do you have a refrigerator?	1. Yes 2. No	
116	Do you have a laundry machine?	1. Yes 2. No	
117	Do you have a bicycle/motorcycle?	1. Yes 2. No	
118	Do you have a car?	1. Yes 2. No	
119	Do you have a domestic servant?	1. Yes 2. No	
120	Homeownership?	1. Private<br>2. Government<br>3. Rent<br>4. Other ————	
121	How many rooms are in your home?	____________	
122	How many individuals are there per sleeping room?	______________	
123	Which type of roofing material is used?	1. Natural material<br>2. Corrugated iron<br>3. Tiles<br>4. Other————	
124	Which type of flooring material is used?	1. Mud<br>2. Parquet/polished wood<br>3. Cement<br>4. Ceramic tiles<br>5. Carpet<br>6. Other ————	

<u>Part 2: Questions related to reproductive health factors.</u>

201	Do you ever use contraceptives?	1. Yes<br>2. No	If No, go to 206
202	Have you ever used oral Contraceptives?	1. Yes<br>2. No	
203	If yes, Q202 for how long?	_________________	
204	Have you ever used IUCD?	1. Yes<br>2. No	
205	If yes, Q204 for how long?	______________	

206	How old were you when your menarche?	__________________	
207	How was your menstrual history?	1. Regular<br>2. Same times irregular<br>3. Always irregular<br>4. No menses. Why? ____________	
208	Have you ever experienced post-coital bleeding?	1. Yes<br>2. No	
209	Have you ever given birth?	1. Yes<br>2. No	If no, go to 215
210	If yes, for Q209, how many?	______________	
211	How old were you when you first gave birth?	_________________	
212	Have you ever experienced genital trauma during delivery?	1. Yes<br>2. No	
213	If the answer for Q212yes, which type of trauma	1. Cervix<br>2. Vagina<br>3. Both	
214	What is the average birth interval between your births? (if she has two or more births)	___________	
215	Have you ever –experienced abortion?	1. Yes<br>2. No	
216	If the answer for Q215 is yes, how many money times?	_____________	
217	Do you have a family (mother or sister) history of cervical cancer?	1. Yes<br>2. No	

Part 3: Questions related to lifestyle and sexual behavioural factors.

301	Have you ever been screened for cervical cancer before?	1. Yes<br>2. No	If no, go to 304
302	If the answer for Q301 is yes, when were you screened for the last time?	______________	
303	What was the result of the screening test?	1. Positive<br>2. Negative	
304	Have you ever smoked?	1. Yes<br>2. No	

305	If the answer for Q304 is yes, how long have you been smoking?	________________	
306	How old were you when you first had sex?	_________________	
307	Do you use a condom whenever you are having sex?	1. Yes<br>2. No	
308	Have you ever been told you had a pelvic infection or been treated by health professionals?	1. Yes<br>2. No	
309	Have you had a sexually transmitted infection in your lifetime?	1. Yes<br>2. No	
310	Does your partner ever have a history of STIs?	1. Yes<br>2. No	
311	Do you ever have a history of genital ulcer or swelling?	1. Yes<br>2. No	
312	Does your partner ever have a history of genital ulcer or swelling?	1. Yes<br>2. No	
313	Have you been tested for HIV before?	1. Yes<br>2 . No	
314	If the answer for Q313 is yes, what was the result?	1. Positive<br>2. Negative<br>3. Unknown	
315	If the answer for Q314 is positive, did you start antiretroviral therapy?	1. Yes<br>2. No	
316	How many sexual partners have you had in your lifetime?	_______________	
317	Does your partner have other partners?	1. Yes<br>2. No	
318	If the answer for Q 317 is yes, how many?	_________________	

Part 4 Knowledge of pre-cervical cancer lesions.

401	Have you ever heard about pre-clinical cancer?	1. Yes<br>2. No	
402	If yes, for Q.401, where did you get the information for the first time?	1. News, Media<br>2. Brochures, posters, and other printed materials<br>3. Health workers<br>4. Family, friends, neighbours, and colleagues<br>5. Religious leaders<br>6. Teachers<br>7.Other(please explain):	
403	Do you know the possible cause of pre-cervical cancer?	1 . Yes 2. No	

404	If she says yes to Q number 403, what are the causes?	1, HPV<br>2. Smoking<br>3. Early sexual intercourse<br>4. Family history<br>5. I don't know	
405	Do you know the symptoms of pre-cervical cancer?	1 . Yes 2. No	
406	If yes, for Q, 405, which one is known?	1. Vaginal bleeding<br>2. Post-coital bleeding.<br>3. Vaginal discharges<br>4. Pain during sexual intercourse	
407	Any reproductive-age woman is susceptible to developing pre-cervical cancer?	1 . Yes 2. No	
408	Pre-cervical cancer is more common in women who are HIV Positive.	1 . Yes 2. No	
409	Can you mention any of the procedures used in screening for pre-cervical cancer?	1. VIA<br>2. Pap Smear<br>3. Biopsy<br>4. Don't know	
410	How can a person prevent getting pre-cervical cancer? (Please check all that are mentioned.)	1. Avoid multiple sexual partners<br>2. Avoid early sexual intercourse<br>3. Quit smoking<br>4. Through vaccination, the HPV vaccine<br>5. Do not know<br>6. Other (please explain)	

Part 5: Physical Activity

501	Does your work involve vigorous-intensity physical activity for at least 10 minutes continuously? Like (Jogging, Swimming, walking, basketball, soccer, dancing, cooking, cleaning, home repair, and gardening. Running, carrying heavy groceries or other loads upstairs, shoveling snow by hand, mowing grass with a hand-push mower)	1. Yes<br>2.No	If no, go to p4
502	How many days do you do vigorous-intensity physical activities in a typical week as part of your work? Such as swimming, walking, basketball, soccer, dancing, cooking, cleaning, home repair, and gardening. Running, carrying	____________Number of days	

	heavy groceries or other loads upstairs, shoveling snow by hand, mowing grass with a hand-push mower).		
503	How much time do you spend doing vigorous-intensity activities at work on a typical day?	Hours: minutes └─┴─┘ : └─┴─┘<br>hrs. min	
504	Does your work involve moderate-intensity activity for at least 10 minutes continuously?<br>Like Brisk walking, bike riding, running, jumping rope, and swimming. walking briskly, mopping or vacuuming, or raking a yard	1. Yes<br>2.no	If no, go top 7
505	How often do you do moderate-intensity activities in a typical week as part of your work?	______Number of days	
506	How much time do you spend doing moderate-intensity activities at work on a typical day?	Hours: minutes └─┴─┘ : └─┴─┘<br>hrs. min	
507	Do you continuously walk or use a bicycle for at least 10 minutes to get to and from places?	1. Yes<br>2.No	*If No, go to P 10*
508	In a typical week, how many days do you walk or bicycle	____________Number of days	
509	How much time do you spend walking or bicycling for travel on a typical day?	Hours: minutes └─┴─┘ : └─┴─┘<br>hrs mins	
510	How much time do you usually spend sitting or reclining on a typical day?	Hours: minutes └─┴─┘ : └─┴─┘<br>hrs. mins	

Part 6: Dietary information (Food frequency questionnaire)

The following questions ask about the diet that you usually eat. As you answer these questions, please consider the foods you consumed last month.

No.	Food items	Frequency of consumption							
		Once a day	More than twice a day	Once a week	2-4 times/ week	once a month	1-3 times /month	Never	Skip
601	Bread and cereals								
602	Vegetables								
603	Fruits								
604	Milk, cheese and yoghurt								
605	Meat								
606	Egg								
607	Legumes, nuts, and seeds (E.g. beans, peas, lentils, nuts)								
608	Sugar and sweet foods (E.g. sugar, honey, chocolates, candies, cookies, and cakes)								
609	Sugar drinks like sweetened juice and soft drinks								
610	Oil and fat (E.g. oil, fat or butter added to foods used for cooking)								
611	Fast foods (E.g. Chips, sandwiches, doughnuts, pasty, burgers, pizza, fried foods, and ice cream)								
